# Childhood trauma and comorbid anxiety disorders in patients with bipolar disorder

**DOI:** 10.1192/j.eurpsy.2022.1020

**Published:** 2022-09-01

**Authors:** D. Bougacha, S. Ellouze, R. Jenhani, R. Ghachem

**Affiliations:** 1 Razi hospital, B, Manouba, Tunisia; 2 Hedi Chaker University Hospital, Psychiatry, Sfax, Tunisia

**Keywords:** Childhood Trauma, bipolar disorder, Anxiety disorders

## Abstract

**Introduction:**

A history of childhood trauma and Comorbid anxiety disorders have each been identified as potential predictors of unfavorable outcomes in patients with bipolar disorder. Nevertheless, the relationship between these two prognostic features has been little studied.

**Objectives:**

In the present study, we aim to explore the relationship between childhood trauma and comorbid anxiety disorders in bipolar patients.

**Methods:**

We conducted a cross-sectional, descriptive, and analytical study. Sixty-one euthymic patients with bipolar disorder were recruited in the department of psychiatry B of Razi Hospital, during their follow-up. We assessed history of childhood traumatic experiences with the Childhood Trauma Questionnaire (CTQ) and current diagnosis of anxiety disorders with the M.I.N.I. International Neuropsychiatric Interview.

**Results:**

The mean age of patients was 43.4. The sex ratio was 2.4. Almost two-thirds of patients (64%) had experienced at least one type of childhood trauma. Twenty-one percent of participants had one anxiety disorder and 12% participants had two or more current anxiety disorders. Of the anxiety disorders, social anxiety disorder was significantly associated with emotional abuse subscale (p=0.002). Generalized anxiety disorder was significantly associated with the physical abuse subscale (p=0.025) and the number of severe childhood trauma per patient (p=). A statistically significant association was found between the number of current anxiety disorders and the emotional abuse sub score (p=0.021).

**Conclusions:**

Exposure to childhood traumatic experiences is associated with more common comorbid anxiety disorders among bipolar patients. These prognostic features should systematically be a part of clinical assessment and taken into account in the management of these patients.

**Disclosure:**

No significant relationships.

